# Tall height and obesity are associated with an increased risk of aggressive prostate cancer: results from the EPIC cohort study

**DOI:** 10.1186/s12916-017-0876-7

**Published:** 2017-07-13

**Authors:** Aurora Perez-Cornago, Paul N. Appleby, Tobias Pischon, Konstantinos K. Tsilidis, Anne Tjønneland, Anja Olsen, Kim Overvad, Rudolf Kaaks, Tilman Kühn, Heiner Boeing, Annika Steffen, Antonia Trichopoulou, Pagona Lagiou, Maria Kritikou, Vittorio Krogh, Domenico Palli, Carlotta Sacerdote, Rosario Tumino, H. Bas Bueno-de-Mesquita, Antonio Agudo, Nerea Larrañaga, Elena Molina-Portillo, Aurelio Barricarte, Maria-Dolores Chirlaque, J. Ramón Quirós, Pär Stattin, Christel Häggström, Nick Wareham, Kay-Tee Khaw, Julie A. Schmidt, Marc Gunter, Heinz Freisling, Dagfinn Aune, Heather Ward, Elio Riboli, Timothy J. Key, Ruth C. Travis

**Affiliations:** 1 Cancer Epidemiology Unit, Nuffield Department of Population Health, University of Oxford, Oxford, OX3 7LF United Kingdom; 2 Molecular Epidemiology Group, Max Delbrueck Center for Molecular Medicine in the Helmholtz Association (MDC), Berlin, Germany; 3 Department of Hygiene and Epidemiology, University of Ioannina School of Medicine, Ioannina, Greece; 4 Department of Epidemiology and Biostatistics, School of Public Health, Imperial College London, London, United Kingdom; 5 Danish Cancer Society Research Center, Copenhagen, Denmark; 6 Department of Public Health, Section for Epidemiology, Aarhus University, Aarhus, Denmark; 7 Division of Cancer Epidemiology, German Cancer Research Center (DKFZ), Heidelberg, Germany; 8 Department of Epidemiology, German Institute of Human Nutrition Potsdam-Rehbrücke, Nuthetal, Germany; 9 Hellenic Health Foundation, Athens, Greece; 10 WHO Collaborating Center for Nutrition and Health, Unit of Nutritional Epidemiology and Nutrition in Public Health, Department of Hygiene, Epidemiology and Medical Statistics, University of Athens Medical School, Athens, Greece; 11 Department of Epidemiology, Harvard School of Public Health, Boston, USA; 12 Epidemiology and Prevention Unit, Fondazione IRCCS Istituto Nazionale dei Tumori, Milano, Italy; 13 Cancer Risk Factors and Life-Style Epidemiology Unit, Cancer Research and Prevention Institute – ISPO, Florence, Italy; 14 Unit of Cancer Epidemiology, AO Citta’ della Salute e della Scienza-University of Turin and Center for Cancer Prevention (CPO-Piemonte), Turin, Italy; 15 Cancer Registry and Histopathology Unit, “Civic - M.P. Arezzo” Hospital, Azienda Sanitaria Provinciale, Ragusa, Italy; 16 Department for Determinants of Chronic Diseases (DCD), National Institute for Public Health and the Environment (RIVM), Bilthoven, The Netherlands; 17 Department of Social & Preventive Medicine, Faculty of Medicine, University of Malaya, Kuala Lumpur, Malaysia; 18 Unit of Nutrition and Cancer, Cancer Epidemiology Research Program, Catalan Institute of Oncology-IDIBELL, L’Hospitalet de Llobregat, Barcelona, Spain; 19 Public Health Division of Gipuzkoa, Regional Government of the Basque Country, Donostia, Spain; 20 CIBER of Epidemiology and Public Health (CIBERESP), Madrid, Spain; 21 Escuela Andaluza de Salud Pública, Instituto de Investigación Biosanitaria ibs, GRANADA, Hospitales Universitarios de Granada/Universidad de Granada, Granada, Spain; 22 Navarra Public Health Institute, Pamplona, Spain; 23 Navarra Institute for Health Research (IdiSNA), Pamplona, Spain; 24 Department of Epidemiology, Regional Health Council, IMIB-Arrixaca, Murcia, Spain; 25 Department of Health and Social Sciences, Universidad de Murcia, Murcia, Spain; 26 Public Health Directorate, Asturias, Spain; 27 Department of Surgical Sciences, Uppsala University, Uppsala, Sweden; 28 Department of Surgical and Perioperative Sciences, Urology and Andrology, Umeå University, Umeå, Sweden; 29 Department of Biobank Research, Umeå University, Umeå, Sweden; 30 MRC Epidemiology Unit, University of Cambridge, Cambridge, United Kingdom; 31 University of Cambridge School of Clinical Medicine, Cambridge, United Kingdom; 32 Section of Nutrition and Metabolism, International Agency for Research on Cancer, Lyon, France

**Keywords:** Adiposity, Obesity, Height, Prostate cancer, Cohort study, Tumour characteristics, High grade

## Abstract

**Background:**

The relationship between body size and prostate cancer risk, and in particular risk by tumour characteristics, is not clear because most studies have not differentiated between high-grade or advanced stage tumours, but rather have assessed risk with a combined category of aggressive disease. We investigated the association of height and adiposity with incidence of and death from prostate cancer in 141,896 men in the European Prospective Investigation into Cancer and Nutrition (EPIC) cohort.

**Methods:**

Multivariable-adjusted Cox proportional hazards models were used to calculate hazard ratios (HRs) and 95% confidence intervals (CIs). After an average of 13.9 years of follow-up, there were 7024 incident prostate cancers and 934 prostate cancer deaths.

**Results:**

Height was not associated with total prostate cancer risk. Subgroup analyses showed heterogeneity in the association with height by tumour grade (*P*_heterogeneity_ = 0.002), with a positive association with risk for high-grade but not low-intermediate-grade disease (HR for high-grade disease tallest versus shortest fifth of height, 1.54; 95% CI, 1.18–2.03). Greater height was also associated with a higher risk for prostate cancer death (HR = 1.43, 1.14–1.80). Body mass index (BMI) was significantly inversely associated with total prostate cancer, but there was evidence of heterogeneity by tumour grade (*P*_heterogeneity_ = 0.01; HR = 0.89, 0.79–0.99 for low-intermediate grade and HR = 1.32, 1.01–1.72 for high-grade prostate cancer) and stage (*P*_heterogeneity_ = 0.01; HR = 0.86, 0.75–0.99 for localised stage and HR = 1.11, 0.92–1.33 for advanced stage). BMI was positively associated with prostate cancer death (HR = 1.35, 1.09–1.68). The results for waist circumference were generally similar to those for BMI, but the associations were slightly stronger for high-grade (HR = 1.43, 1.07–1.92) and fatal prostate cancer (HR = 1.55, 1.23–1.96).

**Conclusions:**

The findings from this large prospective study show that men who are taller and who have greater adiposity have an elevated risk of high-grade prostate cancer and prostate cancer death.

**Electronic supplementary material:**

The online version of this article (doi:10.1186/s12916-017-0876-7) contains supplementary material, which is available to authorized users.

## Background

Prostate cancer is the most common cancer in men in Europe, and the second most frequently diagnosed cancer in men worldwide [1]. While relatively little is known about prostate cancer aetiology, hormones have been implicated; for example, circulating insulin-like growth factor I (IGF-I) concentrations are associated with prostate cancer risk [2]. A relatively large body size has been proposed to influence several metabolic and hormonal mechanisms that can promote cancer development [3]. With increasing global rates of overweight and obesity [4], the potential role of obesity in carcinogenesis has become a significant public health concern.

Several prospective studies have investigated the association of body size with the development of prostate cancer [3, 5–11]. The results have suggested differences in associations of body size with risk according to prostate tumour characteristics [12], but relatively few studies have investigated whether body size is related to a higher risk of clinically relevant aggressive prostate cancer [3, 7–9]. The latest World Cancer Research Fund meta-analysis reported that height was related to total prostate cancer and ‘advanced’ prostate cancer, including as ‘advanced’ prostate cancer various aggressive forms of the disease, but not differentiating between stage, grade and prostate cancer death because of the small number of available studies with data on these separate outcomes [12]. This meta-analysis also reported that obesity was associated with ‘advanced’ prostate cancer. The latest meta-analysis published in a peer-reviewed journal classified incident prostate cases into two categories (‘localised’ or ‘advanced’) using a combination of Gleason score, World Health Organization (WHO) grading system, tumour node metastasis (TNM) stage, Jewett–Whitmore staging system and prostate-specific antigen (PSA) levels [13]. Therefore, more studies of the association of body size with prostate cancer separately by both grade and stage are needed.

The current study is an extended analysis of the association between body size and prostate cancer incidence in the European Prospective Investigation into Cancer and Nutrition (EPIC), with an additional 5 years of follow-up (13.9 compared to 8.5 years in the previous publication) and almost three times the number of incident cases (7024 compared to 2446 cases, including 726 compared to 580 high grade, and 1388 compared to 499 advanced stage cases), and a substantial number of deaths from prostate cancer (*n* = 934) [14]. Herein, we sought to examine the association of height and adiposity at baseline with both prostate cancer risk by tumour characteristics and with prostate cancer death.

## Methods

### Study cohort

EPIC is a multicentre prospective cohort study designed to investigate the relationships between diet, lifestyle, environmental factors and cancer risk. All participants gave informed consent, and approval of the study was obtained from the Internal Review Board of the International Agency for Research on Cancer (Lyon, France) and from ethics committees at the participating institutions. The full list of all local ethics committees is provided in Additional file 1: Table S1. The methods of recruitment (questionnaires, anthropometric measurements and blood samples) and study design have been previously described [15]. The EPIC cohort consists of 519,978 participants (of whom approximately 150,000 are men) from 23 centres in 10 European countries. Nearly all EPIC participants are white European. In the present study, we describe data for men from 19 centres in 8 of these countries; no data were available for France, Naples (Italy), Norway, and Utrecht (Netherlands) because these sub-cohorts only included women. Men were not eligible for this analysis if they had previously been registered as having cancer at the time of completing the baseline questionnaire (other than non-melanoma skin cancer), if they had missing dates of prostate cancer diagnosis or follow-up, or if they had no anthropometric data. The study cohort for these analyses comprised 141,896 men.

### Follow-up for prostate cancer incidence and vital status

Follow-up for incident prostate cancer was provided through record linkage to population cancer registries in Denmark, Italy, the Netherlands, Spain, Sweden and the United Kingdom. In Germany and Greece, follow-up was active and a combination of methods was used, including health insurance records, municipality registries, hospital- or physician-based cancer and pathology registries, and active follow-up of study participants and their next of kin; self-reported incident cancers were verified through medical records. Vital status follow-up was collected by record linkage with regional and/or national mortality registries or by active follow-up (in Germany and Greece). A total of 7024 men developed malignant prostate cancer (code: C61) according to the 10th Revision of the International Statistical Classification of Diseases, Injuries and Causes of Death [16].

Data on TNM stage and histological grade were collected from each centre, where possible. Grade was stratified as low-intermediate (Gleason score of < 8 or grade coded as well, moderately or poorly differentiated; *n* = 3749) or high grade (Gleason score of ≥ 8, or grade coded as undifferentiated; *n* = 726) (Additional file 1: Table S2). Advanced stage cases were tumours that had spread beyond the prostate at diagnosis (T_3_–T_4_ and/or N_1_–N_3_ and/or M_1_, and/or stage coded in the recruitment centre as metastatic, n = 1388). Localised stage cases were those confined within the prostate and with no metastases at diagnosis (TNM staging score of ≤ T_2_ and N_0_/N_x_ and M_0_, or stage coded in the recruitment centre as localised, *n* = 2634). Fatal cases were men who died of prostate cancer (*n* = 934).

### Assessment of anthropometrics and other predictor variables

The anthropometry protocols in the EPIC study centres have been previously described in detail [17]. Anthropometric data were directly measured by trained study personnel in most of the participants, but it was self-reported in the majority of participants from EPIC-Oxford, although the accuracy of these self-reported data has been validated [18]. Briefly, weight and height were measured with participants wearing no shoes. Body mass index (BMI) was calculated as weight in kilograms divided by height in metres squared (kg/m^2^). Waist circumference was measured either at the narrowest torso circumference or at the midpoint between the lower ribs and iliac crest or a combination of these methods. Hip circumference was measured at the level of the largest lateral extension of the hips or over the buttocks. To compute the waist to hip ratio (WHR), waist circumference was divided by hip circumference. Each participant’s anthropometric data were corrected for the clothing worn during measurement in order to reduce heterogeneity due to protocol differences between centres [17]. Weight, height, waist circumference and hip circumference were missing for 640 (0.4%), 391 (0.3%), 13,285 (9.3%) and 15,657 (11.0%) participants, respectively, and these individuals were not included in analyses that include these variables.

Baseline data on lifestyle, health status and socio-demographic characteristics were collected via standardised questionnaires, including diet, medical history, lifetime history of tobacco smoking and alcoholic beverage consumption, physical activity [19], marital status, occupational history and level of education [15]. However, screening data were not available in these analyses.

### Statistical analysis

Analyses of the association of anthropometric factors and covariates with prostate cancer risk were conducted by using Cox proportional hazards regression, and hazard ratios (HRs) and 95% confidence intervals (CIs) were calculated. The date of last follow-up ranged from January 2011 in Germany to October 2013 in Spain. Age was used as the underlying time variable and data were stratified by centre and age at recruitment (<50, 50–54.9, 55–59.9, 60–64.9, 65–69.9, and ≥ 70 years) in all models. Entry time was defined as age at recruitment, while exit time was age at censoring (i.e. age at last follow-up, first diagnosis of incident cancer, loss to follow-up or death, whichever came first). Exit time for the analysis of prostate cancer death was age when participants died due to the prostate tumour or censoring (other cause of death, lost to follow-up or end of follow-up period for each centre, whichever was first). To check for violation of the proportional hazards assumption we used time-varying covariates and the Schoenfeld residuals, which indicated no evidence of deviation from the proportional hazards assumption. Potential non-linear associations between the anthropometric variables and prostate cancer risk were evaluated using likelihood ratio tests comparing the model with the anthropometric variable entered as an ordered categorical (ordinal) variable to a nested model with the categorical variable treated as continuous, and no evidence of non-linearity was observed. Tests for linear trend were conducted using continuous values for each anthropometric variable. Multivariable models were adjusted for known or suspected risk factors for prostate cancer, including education level (less than university, university graduate, missing), smoking status (never, former, current, missing), marital status (married, not married, missing), diabetes (yes, no, missing), and physical activity (inactive, moderately inactive, moderately active, active, missing) [20].

The following categories for the main exposure variables were used: (1) height (fifths, and per 10 cm increase); (2) BMI (fifths, per 5 kg/m^2^ increase, and as predefined WHO categories [21] (<25, 25–29.9, and ≥ 30 kg/m^2^)); and (3) waist circumference (fifths, per 10 cm increase, and as predefined WHO categories [22] (<94, 94–101.9, ≥ 102 cm)). The secondary exposure variables were: (1) hip circumference (fifths, and per 10 cm increase) and (2) WHR (fifths, per 0.1 unit increase, and as predefined WHO categories [22] (<0.90, ≥ 0.90)). The fifths were based on fifths of the distribution among non-cases.

Likelihood ratio tests were used to examine the heterogeneity of the associations of the anthropometric variables with risk of prostate cancer categorised according to histological grade (low-intermediate or high grade) and prostate tumour stage (localised or advanced). For this, we fitted stratified Cox models based on competing risks and compared the risk coefficients and standard errors in the subgroups of interest after excluding cases of unknown stage or grade, as appropriate [23].

We also conducted supplementary analyses restricted to high-grade tumours and prostate cancer death to further examine the results from the main analysis. Tests for heterogeneity of trends for the case-defined characteristics (age at diagnosis (<65, ≥ 65 years) and time between blood collection and diagnosis (<5, ≥ 5 years)) were obtained by fitting separate models for each subgroup and assuming independence of the HRs using a competing risk approach. For the non-case-defined factors (e.g. age at recruitment (<60, ≥ 60 years)), the test for heterogeneity was assessed by using a likelihood ratio test to compare the Cox models with and without interaction terms for the anthropometric variable and the relevant factor.

Sensitivity analyses was also performed by excluding extreme values (percentiles outside 1–99); additionally adjusting for total intake of energy, alcohol, fruit and vegetables, red meat, processed meat, protein from dairy sources or height; excluding men with missing values for the main covariates; and using the waist circumference-adjusted residuals of BMI and the BMI-adjusted residuals of waist circumference by regressing these variables in a linear regression model and using the residuals (that are statistically independent of waist circumference or BMI, respectively), as the exposures of interest [24].

Statistical analyses were performed with the Stata 14.0 statistical software package 16. All tests of statistical significance were two-sided and *P* values below 0.05 were considered significant.

## Results

After an average of 13.9 years of follow-up, a total of 7024 men were diagnosed with prostate cancer among the 141,896 men included in this study. Among the total cases, there were 934 deaths from prostate cancer. The mean age at diagnosis was 67.8 years (range, 41–95 years). The main baseline characteristics of the participants according to categories of BMI (<25, 25–29.9 and ≥ 30 kg/m^2^) are shown in Table 1 (and by height and waist circumference in Additional file 1: Tables S3 and S4; the distribution of the study participants and of prostate cancer cases by country is shown in Additional file 1: Table S5). Participants with obesity at baseline were more likely to be older, former smokers, moderately inactive and with a lower level of education than were men of normal BMI. Men with obesity were more likely to be missing information on marital status, but of those men who provided information on marital status at recruitment, men with obesity were more likely to be married.

**Table 1:** Baseline characteristics of male participants according to body mass index (BMI) categories in the European Prospective Investigation into Cancer and Nutrition (EPIC) study

	BMI (kg/m^2^)		
Characteristic	<25	25–29.9	≥30
Number of men	50,678	68,736	21,698
Age at baseline,^a^ years	50.0 (11.2)	52.5 (9.4)	53.3 (8.9)
Age at diagnosis,^a^ years	67.7 (6.9)	68.0 (6.5)	67.7 (6.3)
Smoking status, *N* (%)			
Never smoker	19,156 (37.8)	21,354 (31.1)	5914 (27.3)
Former smoker	15,113 (29.8)	26,916 (39.2)	9162 (42.2)
Current smoker	15,749 (31.1)	19,480 (28.3)	6327 (29.2)
Unknown	660 (1.3)	986 (1.4)	295 (1.4)
Physical activity, *N* (%)			
Inactive	8141 (16.1)	12,998 (18.9)	5309 (24.5)
Moderately inactive	15,452 (30.5)	21,203 (30.8)	6506 (30.0)
Moderately active	12,597 (24.9)	16,636 (24.2)	5046 (23.3)
Active	13,135 (25.9)	16,487 (24.0)	4539 (20.9)
Unknown	1353 (2.7)	1412 (2.1)	298 (1.4)
Diabetes at baseline, *N* (%)			
No	48,380 (95.5)	64,886 (94.4)	19,692 (90.8)
Yes	1088 (2.1)	2424 (3.5)	1542 (7.1)
Unknown	1210 (2.4)	1426 (2.1)	464 (2.1)
Education, *N* (%)			
Below degree level	32,488 (64.1)	50,091 (72.9)	17,485 (80.6)
Degree level	16,644 (32.8)	16,919 (24.6)	3730 (17.2)
Unknown	1546 (3.1)	1726 (2.5)	483 (2.2)
Marital status, *N* (%)			
Married	29,318 (57.9)	38,540 (56.1)	11,029 (50.8)
Not married	9154 (18.1)	7209 (10.5)	2099 (9.7)
Unknown	12,206 (24.1)	22,987 (33.4)	8570 (39.5)
Height,^a^ cm	176.2 (7.2)	174.3 (7.2)	172.6 (7.6)
BMI,^a^ kg/m^2^	23.0 (1.6)	27.2 (1.4)	32.6 (2.7)
Weight,^a^ kg	71.3 (7.3)	82.7 (7.7)	97.3 (11.4)
Waist circumference,^a^ cm	85.8 (6.2)	96.2 (6.2)	109.0 (8.2)
Hip circumference,^a^ cm	95.6 (4.6)	101.6 (4.7)	109.7 (6.6)
Waist to hip ratio^a^	0.899 (0.056)	0.948 (0.054)	0.995 (0.057)

^a^Values are means (SD)

The relationship of height with prostate cancer risk is shown in Table 2. When we compared the highest fifth with the lowest, height was not associated with total prostate cancer risk (HR = 1.06, 95% CI, 0.97–1.15; *P*_trend_ = 0.3). There was evidence of heterogeneity by tumour grade (*P*_heterogeneity_ = 0.002), with height being positively associated with high-grade disease (HR = 1.54, 1.18–2.03; *P*_trend_ = 0.006), but not low-intermediate-grade disease (HR = 0.96, 0.86–1.08; *P*_trend_ = 0.2). Taller height was also associated with a higher risk for prostate cancer death (HR = 1.43, 1.14–1.80; *P*_trend_ = 0.001). The risks of high-grade disease and prostate cancer death increased by 21% (HR = 1.21, 1.06–1.38) and 17% (HR = 1.17, 1.04–1.31), respectively, with every 10 cm increment in height.

**Table 2:** Multivariable-adjusted hazard ratios (95% CI) for prostate cancer in relation to height at recruitment in men from the European Prospective Investigation into Cancer and Nutrition (EPIC) study

	No. of cases	Fifths							Continuous per 10 cm
		1	2	3	4	5			
Height, cm		HR	HR (95% CI)	HR (95% CI)	HR (95% CI)	HR (95% CI)	*P* for trend^1^	*P* for het.^2^	
Median (range)		165.2 (115.0–168.5)	171.0 (168.5–173.0)	175.0 (173.0–176.7)	178.9 (176.7–181.0)	184.2 (181.0–210.0)			
Total prostate cancer	7010	1.00 ref	1.04 (0.96–1.12)	1.02 (0.94–1.10)	1.02 (0.94–1.10)	1.06 (0.97–1.15)	0.3		1.02 (0.98–1.06)
Grade									
Low-intermediate	3740	1.00 ref	0.96 (0.87–1.06)	0.97 (0.88–1.08)	0.91 (0.82–1.01)	0.96 (0.86–1.08)	0.2		0.97 (0.92–1.03)
High	726	1.00 ref	1.30 (1.02–1.66)	1.39 (1.08–1.79)	1.35 (1.04–1.74)	1.54 (1.18–2.03)	0.006	0.002	1.21 (1.06–1.38)
Stage									
Localised	2632	1.00 ref	1.02 (0.91–1.15)	1.01 (0.89–1.14)	0.93 (0.82–1.06)	1.01 (0.88–1.16)	0.7		0.98 (0.92–1.05)
Advanced	1387	1.00 ref	1.04 (0.88–1.24)	1.11 (0.92–1.32)	1.13 (0.95–1.35)	1.09 (0.90–1.32)	0.4	0.2	1.06 (0.96–1.16)
Prostate cancer deaths	932	1.00 ref	1.13 (0.92–1.39)	1.17 (0.94–1.46)	1.13 (0.91–1.41)	1.43 (1.14–1.80)	0.001		1.17 (1.04–1.31)

Cox regression analysis. All models are stratified by centre and age at recruitment and adjusted for education level (less than university, university graduate, missing), smoking status (never, former, current, missing), marital status (married, not married, missing), diabetes (yes, no, missing), and physical activity (inactive, moderately inactive, moderately active, active, missing)

^1^ *P* values for trend are obtained by entering the continuous height in the model

^2^ *P* value from test for heterogeneity for the associations of height with risk of prostate cancer categorised according to prostate tumour grade (low-intermediate or high) and stage (localised or advanced)

Low-intermediate grade (Gleason score of < 8, or grade coded as well, moderately, or poorly differentiated). High grade (Gleason score of ≥ 8, or grade coded as undifferentiated). Localised stage (TNM staging score of ≤ T_2_ and N0/Nx and M0, or stage coded in the recruitment centre as localised). Advanced stage (T_3_–T_4_ and/or N_1_–N_3_ and/or M1, and/or stage coded in the recruitment centre as metastatic)

Total prostate cancer risk was inversely related to BMI and waist circumference (Table 3); the HRs for highest fifth versus lowest were 0.90 (0.83–0.97, *P*_trend_ < 0.001) for BMI and 0.92 (0.84–1.00, *P*_trend_ = 0.01) for waist circumference. However, the association of BMI and waist circumference with prostate cancer risk was found to differ between different prostate cancer tumour characteristics. For BMI and prostate cancer risk, there was evidence of heterogeneity by tumour grade (*P*_heterogeneity_ = 0.01; HR = 0.89, 0.79–0.99 for low-intermediate-grade and HR = 1.32, 1.01–1.72 for high-grade cancer) and stage (*P*_heterogeneity_ = 0.01; HR = 0.86, 0.75–0.99 for localised stage and HR = 1.11, 0.92–1.33 for advanced stage). Similarly, there was significant heterogeneity in the association with waist circumference by tumour grade (*P*_heterogeneity_ = 0.002; HR = 0.87, 0.77–0.99 for low-intermediate-grade and HR = 1.43, 1.07–1.92 for high-grade cancer), but not by tumour stage (*P*_heterogeneity_ = 0.1). There were statistically significant positive associations of prostate cancer death with BMI (HR = 1.35, 1.09–1.68) and waist circumference (HR = 1.55, 1.23–1.96).

**Table 3:** Multivariable-adjusted hazard ratios (95% CI) for prostate cancer in relation to body mass index (BMI) and waist circumference at recruitment in men from the European Prospective Investigation into Cancer and Nutrition (EPIC) study

	No. of cases	Fifths					*P* for trend^1^	*P* for het.^2^	
		1	2	3	4	5			
		HR	HR (95% CI)	HR (95% CI)	HR (95% CI)	HR (95% CI)			
BMI, kg/m^2^									Continuous per 5 kg/m^2^
Median (range)		22.2 (12.7–23.5)	24.5 (23.5–25.3)	26.1 (25.3–27.0)	28.0 (27.0–29.2)	31.1 (29.2–68.4)			
Total prostate cancer	6991	1.00 ref	0.99 (0.92–1.07)	1.04 (0.96–1.12)	0.94 (0.87–1.01)	0.90 (0.83–0.97)	<0.001		0.94 (0.90–0.98)
Grade									
Low-intermediate	3727	1.00 ref	0.98 (0.88–1.09)	1.08 (0.97–1.20)	0.90 (0.80–1.00)	0.89 (0.79–0.99)	0.001		0.92 (0.87–0.98)
High	720	1.00 ref	1.38 (1.07–1.77)	1.30 (1.01–1.68)	1.32 (1.02–1.71)	1.32 (1.01–1.72)	0.07	0.01	1.10 (0.97–1.25)
Stage									
Localised	2622	1.00 ref	1.03 (0.91–1.17)	1.00 (0.88–1.14)	0.91 (0.80–1.03)	0.86 (0.75–0.99)	0.001		0.90 (0.84–0.97)
Advanced	1384	1.00 ref	1.04 (0.87–1.24)	1.24 (1.05–1.48)	1.08 (0.90–1.29)	1.11 (0.92–1.33)	0.3	0.01	1.05 (0.96–1.15)
Prostate cancer deaths	931	1.00 ref	1.15 (0.93–1.42)	1.20 (0.97–1.48)	1.05 (0.84–1.31)	1.35 (1.09–1.68)	0.01		1.14 (1.02–1.27)
Waist, cm									Continuous per 10 cm
Median (range)		82.5 (51.0–86.0)	89.0 (86.0–91.9)	94.0 (91.9–96.5)	99.5 (96.5–103.0)	108.0 (103.0–180.0)			
Total prostate cancer	6352	1.00 ref	1.01 (0.93–1.09)	1.00 (0.93–1.09)	0.99 (0.91–1.07)	0.92 (0.84–1.00)	0.01		0.97 (0.94–1.00)
Grade									
Low-intermediate	3251	1.00 ref	0.97 (0.87–1.09)	0.97 (0.86–1.09)	0.95 (0.85–1.06)	0.87 (0.77–0.99)	0.003		0.95 (0.91–0.99)
High	641	1.00 ref	1.36 (1.02–1.81)	1.26 (0.94–1.67)	1.58 (1.20–2.08)	1.43 (1.07–1.92)	0.001	0.002	1.13 (1.03–1.25)
Stage									
Localised	2075	1.00 ref	0.96 (0.82–1.11)	0.96 (0.83–1.11)	0.93 (0.80–1.08)	0.89 (0.76–1.03)	0.03		0.96 (0.91–1.01)
Advanced	1291	1.00 ref	1.20 (1.00–1.46)	1.17 (0.97–1.41)	1.30 (1.08–1.56)	1.08 (0.89–1.32)	0.2	0.1	1.03 (0.96–1.10)
Prostate cancer deaths	877	1.00 ref	1.21 (0.96–1.53)	1.03 (0.81–1.31)	1.28 (1.02–1.61)	1.55 (1.23–1.96)	<0.001		1.18 (1.08–1.28)

Cox regression analysis. All models are stratified by centre and age at recruitment and adjusted for education level (less than university, university graduate, missing), smoking status (never, former, current, missing), marital status (married, not married, missing), diabetes (yes, no, missing), and physical activity (inactive, moderately inactive, moderately active, active, missing)

^1^ *P* values for trend are obtained by entering the continuous anthropometric variable in the model

^2^ *P* value from test for heterogeneity for the associations of the anthropometric variable with risk of prostate cancer categorised according to prostate tumour grade (low-intermediate or high) and stage (localised or advanced)

Low-intermediate grade (Gleason score of < 8, or grade coded as well, moderately, or poorly differentiated). High grade (Gleason score of ≥ 8, or grade coded as undifferentiated). Localised stage (TNM staging score of ≤ T_2_ and N0/Nx and M0, or stage coded in the recruitment centre as localised). Advanced stage (T_3_–T_4_ and/or N_1_–N_3_ and/or M1, and/or stage coded in the recruitment centre as metastatic)

The associations of hip circumference and WHR with prostate cancer risk are shown in Additional file 1: Table S6. Total prostate cancer was inversely associated with hip circumference (HR for highest versus lowest fifth 0.86, 0.79–0.94). There was significant heterogeneity for hip circumference by tumour grade (*P*_heterogeneity_ < 0.001; HR = 0.84, 0.75–0.95 for low-intermediate-grade and HR = 1.37, 1.04–1.80 for high-grade cancer). WHR was not associated with total prostate cancer incidence. However, there was evidence of heterogeneity by cancer grade (*P*_heterogeneity_ = 0.004) and stage (*P*_heterogeneity_ = 0.02); WHR was positively associated with high-grade (HR = 1.46, 1.09–1.94, *P*_trend_ = 0.004) and advanced stage (HR = 1.29, 1.05–1.58, *P*_trend_ = 0.01), but not with low-intermediate-grade and localised prostate cancer. Hip circumference was significantly associated with risk of death from prostate cancer (HR for highest versus lowest fifth 1.43, 1.14–1.79), but no association between WHR and prostate cancer death was observed.

When BMI, waist circumference and WHR were categorised according to the WHO cut-off points, the results were broadly similar to those for these variables categorised in fifths (Additional file 1: Table S7).

There was no evidence of heterogeneity for the associations of height, BMI and waist circumference with high-grade prostate cancer and prostate cancer death by age at recruitment (<60, ≥ 60 years), age at diagnosis (<65, ≥ 65 years), or time between recruitment and diagnosis (<5, ≥ 5 years) (Additional file 1: Tables S8 (height), S9 (BMI) and S10 (waist circumference)).

The significant positive association of height, BMI and waist circumference with high-grade prostate cancer and prostate cancer death remained largely unchanged in the sensitivity analysis. After excluding men with missing data for covariates, we observed that the association of waist circumference with both high-grade prostate cancer and prostate cancer death was slightly larger, although the associations of height and BMI with risk were somewhat attenuated and were no longer statistically significant. When we used waist circumference-adjusted residuals of BMI as the exposure, the positive association between BMI and prostate cancer death was no longer statistically significant and the direction was reversed (HR = 0.80, 0.51–1.25). When we used BMI-adjusted residuals of waist circumference as the exposure the association between waist circumference and prostate cancer death was essentially unchanged.

## Discussion

In this prospective analysis, men who were taller and with greater adiposity had an elevated risk of high-grade prostate cancer and death from prostate cancer. The associations were strongest for height and waist circumference.

Previous studies have found a positive association between height and risk of prostate cancer [3, 5, 12, 25–30]. In our study, this positive association was only significant for high-grade prostate cancer and death from prostate cancer. While several prospective studies have found a positive association between height and death from prostate cancer [3, 27, 28], to our knowledge, no previous study has found a positive association between height and high-grade prostate cancer risk [3, 29, 30]. However, this might be due to the fact that not many studies have differentiated between prostate cancer stage and grade of the disease [12]. The mechanisms underlying this association of height with aggressive disease are not fully understood. Height is partly determined by genetic factors and it might also be a marker of cumulative early-life growth factor exposures, such as high IGF-I or childhood nutrition, which may increase the risk of prostate cancer [2]. Taller men have more cells (including stem cells) and larger prostate volumes [31]. However, men with smaller prostates have been found to have more high-grade/advanced disease and higher progression rates [32].

Results from previous prospective studies have suggested that the association between obesity and prostate cancer may vary significantly across tumour characteristics [3, 6–9], with a positive association between adiposity and risk of aggressive (advanced stage and high grade combined) prostate cancer [3, 6–9]. The association between genetically determined adiposity and prostate cancer risk has also recently been examined in a large Mendelian randomisation study [33]. No association was observed between genetic score for adult BMI and WHR for total and aggressive prostate cancer (defined as a Gleason score of ≥ 8, a disease stage of ‘distant’, a PSA level of > 100 ng/mL or death from prostate cancer); however, associations were not examined separately by tumour stage and grade [33]. In our analysis, adiposity tended to be positively associated with risk for high-grade tumours and prostate cancer death, and inversely related to non-aggressive prostate cancer tumours and total prostate cancer, which is in accordance with previous reports [9, 11, 34]. The association of obesity with death might be for a specific tumour subtype such as tumours with the TMPRSS2:ERG gene fusion [35]. BMI adjusted for waist circumference probably reflects lean body mass rather than adiposity. The positive association between waist circumference and prostate cancer death was the only one maintained in all of the multiple sensitivity analyses. Although waist circumference and BMI are highly correlated, waist circumference has been proposed as a better marker of adiposity for men [36].

In our analysis, adiposity markers tended to be positively associated with aggressive tumours and prostate cancer mortality, and inversely related to non-aggressive prostate cancer tumours and total prostate cancer. These different associations by tumour characteristics may be partly due to differences in prostate cancer detection in men with obesity. Such men may be less likely to be diagnosed with prostate cancer overall, and in particular with early prostate cancer, because they have lower PSA concentrations (perhaps due to an increased blood volume since the total amount of PSA in blood does not differ by body mass), are less likely to undergo a biopsy, and are also likely to have larger prostates, making cancer detection more difficult [37]. It may be also more difficult to perform a thorough digital rectal examination on men with obesity. A reduced likelihood of early detection and treatment might in turn lead to an elevated incidence of aggressive disease and high prostate cancer mortality in men with obesity [38]. However, several possible biological mechanisms that may underlie the association between obesity and prostate cancer death have been proposed, including mechanisms that involve insulin and the IGF-I axis, sex hormones, and inflammatory and oxidative stress pathways. Obesity is associated with disturbances in the IGF-I axis; an inverted U-shaped association between BMI and IGF-I has been observed, while BMI and concentrations of IGFBP-1 and -2 have been shown to be inversely associated [39]. High circulating IGF-I levels are associated with an increased prostate cancer incidence in this cohort [40] and in an individual participant meta-analysis of prospective studies [2]. Participants with obesity normally suffer from hyperinsulinemia, which has been linked to prostate cancer risk [41] and prostate cancer mortality [42]. Obesity is also related to a decrease in free testosterone and changes in other sex hormone concentrations [38]. Moreover, excess adiposity may contribute to the activation of proinflammatory signalling pathways [43] and higher oxidative stress [44], both of which have been suggested to be linked to higher prostate cancer risk [45, 46].

This study has several strengths and also some limitations. The strengths include its prospective design, detailed information on potential confounders, long follow-up, the large sample size and number of incident cases, and the availability of data on prostate cancer tumour characteristics and mortality. Analyses by time to diagnosis showed no evidence that the observed associations were due to reverse causality. Although measurements of fat mass were not available in this study, previous investigations have shown that both BMI and waist circumference are strongly correlated with total fat mass [47]. Participants in this cohort might be considered to be late middle-aged adults, since their mean age at recruitment was 52 years. In this age group, and especially in older adults, the use of BMI as a measure of overweight and obesity might be less sensitive because ageing is associated with a decrease in muscle mass and height [48]. It might therefore be argued that the use of BMI in our cohort may lead to an underestimate in the prevalence of obesity; however, we also use waist circumference, which has been found to be a better predictor of total body fat, and especially of abdominal adiposity in men, than BMI, because waist circumference is less influenced by muscle mass [48]. The lack of screening data is a limitation of this analysis. Information on tumour characteristics was only available for a subset of cases (24.8% of prostate cancer cases did not have any data on tumour characteristics) and men with obesity were less likely to have missing data on tumour stage and grade than were men who were not overweight. Data on early-life factors, including anthropometry, which may influence prostate cancer occurrence [26, 49], were not available in the current study.

## Conclusion

In summary, the findings from this large European prospective study provide evidence that men with greater height and adiposity (high BMI and waist circumference) have an elevated risk of high-grade prostate cancer and prostate cancer death. The data presented illustrate the complex association of adiposity and prostate cancer, which varies by disease aggressiveness.

## Additional file

Additional file 1: Table S1. List of all of the local ethics committees for the European Prospective Investigation into Cancer and Nutrition (EPIC) study. **Table S2.** Distribution of cases by tumour grade in men from the EPIC study. **Table S3.** Baseline characteristics of participants according to fifths of height at recruitment in men from the EPIC study. **Table S4.** Baseline characteristics of participants according to fifths of waist circumference at recruitment in men from the EPIC study. **Table S5.** Distribution of study participants and prostate cancer cases by country. **Table S6.** Multivariable-adjusted hazard ratios (95% CI) for prostate cancer in relation to hip circumference and waist to hip ratio (WHR) at recruitment in men from the EPIC study. **Table S7.** Multivariable-adjusted hazard ratios (95% CI) for prostate cancer in relation to BMI, waist circumference and WHR using the WHO cut-off points at recruitment in men from the EPIC study. **Table S8.** Stratified and sensitivity analyses. Multivariable-adjusted hazard ratios (95% CI) for high-grade prostate cancer and death from prostate cancer in relation to height (per 10 cm unit increase) at recruitment in men from the EPIC study. **Table S9.** Stratified and sensitivity analyses. Multivariable-adjusted hazard ratios (95% CI) for high-grade prostate cancer and death from prostate cancer in relation to BMI (per 5 kg/m^2^ unit increase) at recruitment in men from the EPIC study. **Table S10.** Stratified and sensitivity analyses. Multivariable-adjusted hazard ratios (95% CI) for high-grade prostate cancer and death from prostate cancer in relation to waist circumference (per 10 cm unit increase) at recruitment in men from the EPIC study. (DOCX 63 kb)

## Abbreviations

BMI: body mass index
CIs: confidence intervals
EPIC: European Prospective Investigation into Cancer and Nutrition
HRs: hazard ratios
IGF-I: insulin-like growth factor I
PSA: prostate-specific antigen
TNM: tumour node metastasis
WHO: World Health Organization
WHR: waist to hip ratio

## References

[1] Ferlay J, Soerjomataram I, Dikshit R, Eser S, Mathers C, Rebelo M, Parkin DM, Forman D, Bray F (2015). Cancer incidence and mortality worldwide: sources, methods and major patterns in GLOBOCAN 2012. Int J Cancer.

[2] Travis RC, Appleby PN, Martin RM, Holly JM, Albanes D, Black A, Bueno-de-Mesquita HB, Chan JM, Chen C, Chirlaque MD (2016). A meta-analysis of individual participant data reveals an association between circulating levels of IGF-I and prostate cancer risk. Cancer Res.

[3] Moller E, Wilson KM, Batista JL, Mucci LA, Balter K, Giovannucci E (2015). Body size across the life course and prostate cancer in the Health Professionals Follow-up Study. Int J Cancer.

[4] Ng M, Fleming T, Robinson M, Thomson B, Graetz N, Margono C, Mullany EC, Biryukov S, Abbafati C, Abera SF (2014). Global, regional, and national prevalence of overweight and obesity in children and adults during 1980-2013: a systematic analysis for the Global Burden of Disease Study 2013. Lancet.

[5] MacInnis RJ, English DR (2006). Body size and composition and prostate cancer risk: systematic review and meta-regression analysis. Cancer Causes Control.

[6] Cao Y, Ma J (2011). Body mass index, prostate cancer-specific mortality, and biochemical recurrence: a systematic review and meta-analysis. Cancer Prev Res (Phila).

[7] Littman AJ, White E, Kristal AR (2007). Anthropometrics and prostate cancer risk. Am J Epidemiol.

[8] Vidal AC, Howard LE, Moreira DM, Castro-Santamaria R, Andriole GL, Freedland SJ (2014). Obesity increases the risk for high-grade prostate cancer: results from the REDUCE study. Cancer Epidemiol Biomarkers Prev.

[9] Rodriguez C, Freedland SJ, Deka A, Jacobs EJ, McCullough ML, Patel AV, Thun MJ, Calle EE (2007). Body mass index, weight change, and risk of prostate cancer in the Cancer Prevention Study II Nutrition Cohort. Cancer Epidemiol Biomarkers Prev.

[10] Engeland A, Tretli S, Bjorge T (2003). Height, body mass index, and prostate cancer: a follow-up of 950000 Norwegian men. Br J Cancer.

[11] Wright ME, Chang SC, Schatzkin A, Albanes D, Kipnis V, Mouw T, Hurwitz P, Hollenbeck A, Leitzmann MF (2007). Prospective study of adiposity and weight change in relation to prostate cancer incidence and mortality. Cancer.

[12] WCRF/AICR. World Cancer Research Fund International/American Institute for Cancer Research Continuous Update Project Report: Diet, Nutrition, Physical Activity, and Prostate Cancer. 2014. http://www.wcrf.org/sites/default/files/Prostate-Cancer-SLR-2014.pdf. Accessed 1 Apr 2016.

[13] Discacciati A, Orsini N, Wolk A (2012). Body mass index and incidence of localized and advanced prostate cancer--a dose-response meta-analysis of prospective studies. Ann Oncol.

[14] Pischon T, Boeing H, Weikert S, Allen N, Key T, Johnsen NF, Tjonneland A, Severinsen MT, Overvad K, Rohrmann S (2008). Body size and risk of prostate cancer in the European prospective investigation into cancer and nutrition. Cancer Epidemiol Biomarkers Prev.

[15] Riboli E, Hunt KJ, Slimani N, Ferrari P, Norat T, Fahey M, Charrondiere UR, Hemon B, Casagrande C, Vignat J (2002). European prospective investigation into cancer and nutrition (EPIC): study populations and data collection. Public Health Nutr.

[16] World Health Organization. International statistical classification of diseases and related health problems. 10th revision. http://apps.who.int/classifications/icd10/browse/2010/en. Accessed 01 Apr 2016.

[17] Haftenberger M, Lahmann PH, Panico S, Gonzalez CA, Seidell JC, Boeing H, Giurdanella MC, Krogh V, Bueno-de-Mesquita HB, Peeters PH (2002). Overweight, obesity and fat distribution in 50- to 64-year-old participants in the European Prospective Investigation into Cancer and Nutrition (EPIC). Public Health Nutr.

[18] Spencer EA, Roddam AW, Key TJ (2004). Accuracy of self-reported waist and hip measurements in 4492 EPIC-Oxford participants. Public Health Nutr.

[19] Wareham NJ, Jakes RW, Rennie KL, Schuit J, Mitchell J, Hennings S, Day NE (2003). Validity and repeatability of a simple index derived from the short physical activity questionnaire used in the European Prospective Investigation into Cancer and Nutrition (EPIC) study. Public Health Nutr.

[20] InterAct C, Peters T, Brage S, Westgate K, Franks PW, Gradmark A, Tormo Diaz MJ, Huerta JM, Bendinelli B, Vigl M (2012). Validity of a short questionnaire to assess physical activity in 10 European countries. Eur J Epidemiol.

[21] Physical status: the use and interpretation of anthropometry. Report of a WHO Expert Committee. World Health Organ Tech Rep Ser. 1995;854:1–452. 8594834

[22] World Health Organization (2008). Waist Circumference and Waist-hip Ratio: Report of a WHO Expert Consultation.

[23] Smith-Warner SA, Spiegelman D, Ritz J, Albanes D, Beeson WL, Bernstein L, Berrino F, van den Brandt PA, Buring JE, Cho E (2006). Methods for pooling results of epidemiologic studies: the Pooling Project of Prospective Studies of Diet and Cancer. Am J Epidemiol.

[24] Roswall N, Freisling H, Bueno-de-Mesquita HB, Ros M, Christensen J, Overvad K, Boutron-Ruault MC, Severi G, Fagherazzi G, Chang-Claude J (2014). Anthropometric measures and bladder cancer risk: a prospective study in the EPIC cohort. Int J Cancer.

[25] Zuccolo L, Harris R, Gunnell D, Oliver S, Lane JA, Davis M, Donovan J, Neal D, Hamdy F, Beynon R (2008). Height and prostate cancer risk: a large nested case-control study (ProtecT) and meta-analysis. Cancer Epidemiol Biomarkers Prev.

[26] Sutcliffe S, Colditz GA (2013). Prostate cancer: is it time to expand the research focus to early-life exposures?. Nat Rev Cancer.

[27] Batty GD, Kivimaki M, Clarke R, Davey Smith G, Shipley MJ (2011). Modifiable risk factors for prostate cancer mortality in London: forty years of follow-up in the Whitehall study. Cancer Causes Control.

[28] Rodriguez C, Patel AV, Calle EE, Jacobs EJ, Chao A, Thun MJ (2001). Body mass index, height, and prostate cancer mortality in two large cohorts of adult men in the United States. Cancer Epidemiol Biomarkers Prev.

[29] Hernandez BY, Park SY, Wilkens LR, Henderson BE, Kolonel LN (2009). Relationship of body mass, height, and weight gain to prostate cancer risk in the multiethnic cohort. Cancer Epidemiol Biomarkers Prev.

[30] Gong Z, Neuhouser ML, Goodman PJ, Albanes D, Chi C, Hsing AW, Lippman SM, Platz EA, Pollak MN, Thompson IM (2006). Obesity, diabetes, and risk of prostate cancer: results from the prostate cancer prevention trial. Cancer Epidemiol Biomarkers Prev.

[31] Fowke JH, Motley SS, Cookson MS, Concepcion R, Chang SS, Wills ML, Smith JA (2007). The association between body size, prostate volume and prostate-specific antigen. Prostate Cancer Prostatic Dis.

[32] Freedland SJ, Isaacs WB, Platz EA, Terris MK, Aronson WJ, Amling CL, Presti JC, Kane CJ (2005). Prostate size and risk of high-grade, advanced prostate cancer and biochemical progression after radical prostatectomy: a search database study. J Clin Oncol.

[33] Gao C, Patel CJ, Michailidou K, Peters U, Gong J, Schildkraut J, Schumacher FR, Zheng W, Boffetta P, Stucker I (2016). Mendelian randomization study of adiposity-related traits and risk of breast, ovarian, prostate, lung and colorectal cancer. Int J Epidemiol.

[34] Zhong S, Yan X, Wu Y, Zhang X, Chen L, Tang J, Zhao J (2016). Body mass index and mortality in prostate cancer patients: a dose-response meta-analysis. Prostate Cancer Prostatic Dis.

[35] Pettersson A, Lis RT, Meisner A, Flavin R, Stack EC, Fiorentino M, Finn S, Graff RE, Penney KL, Rider JR (2013). Modification of the association between obesity and lethal prostate cancer by TMPRSS2:ERG. J Natl Cancer Inst.

[36] Thomas EL, Parkinson JR, Frost GS, Goldstone AP, Dore CJ, McCarthy JP, Collins AL, Fitzpatrick JA, Durighel G, Taylor-Robinson SD (2012). The missing risk: MRI and MRS phenotyping of abdominal adiposity and ectopic fat. Obesity (Silver Spring).

[37] Banez LL, Hamilton RJ, Partin AW, Vollmer RT, Sun L, Rodriguez C, Wang Y, Terris MK, Aronson WJ, Presti JC (2007). Obesity-related plasma hemodilution and PSA concentration among men with prostate cancer. JAMA.

[38] Allott EH, Masko EM, Freedland SJ (2013). Obesity and prostate cancer: weighing the evidence. Eur Urol.

[39] Crowe FL, Key TJ, Allen NE, Appleby PN, Overvad K, Gronbaek H, Tjonneland A, Halkjaer J, Dossus L, Boeing H (2011). A cross-sectional analysis of the associations between adult height, BMI and serum concentrations of IGF-I and IGFBP-1 -2 and -3 in the European Prospective Investigation into Cancer and Nutrition (EPIC). Ann Hum Biol.

[40] Price AJ, Allen NE, Appleby PN, Crowe FL, Travis RC, Tipper SJ, Overvad K, Gronbaek H, Tjonneland A, Johnsen NF (2012). Insulin-like growth factor-i concentration and risk of prostate cancer: results from the European Prospective Investigation into Cancer and Nutrition. Cancer Epidemiol Biomarkers Prev.

[41] Albanes D, Weinstein SJ, Wright ME, Mannisto S, Limburg PJ, Snyder K, Virtamo J (2009). Serum insulin, glucose, indices of insulin resistance, and risk of prostate cancer. J Natl Cancer Inst.

[42] Bensimon L, Yin H, Suissa S, Pollak MN, Azoulay L (2014). Type 2 diabetes and the risk of mortality among patients with prostate cancer. Cancer Causes Control.

[43] Johnson AR, Milner JJ, Makowski L (2012). The inflammation highway: metabolism accelerates inflammatory traffic in obesity. Immunol Rev.

[44] Marseglia L, Manti S, D’Angelo G, Nicotera A, Parisi E, Di Rosa G, Gitto E, Arrigo T (2015). Oxidative stress in obesity: a critical component in human diseases. Int J Mol Sci.

[45] Paschos A, Pandya R, Duivenvoorden WC, Pinthus JH (2013). Oxidative stress in prostate cancer: changing research concepts towards a novel paradigm for prevention and therapeutics. Prostate Cancer Prostatic Dis.

[46] Sfanos KS, De Marzo AM (2012). Prostate cancer and inflammation: the evidence. Histopathology.

[47] Sun Q, van Dam RM, Spiegelman D, Heymsfield SB, Willett WC, Hu FB (2010). Comparison of dual-energy x-ray absorptiometric and anthropometric measures of adiposity in relation to adiposity-related biologic factors. Am J Epidemiol.

[48] Han TS, Wu FC, Lean ME (2013). Obesity and weight management in the elderly: a focus on men. Best Pract Res Clin Endocrinol Metab.

[49] Kelly SP, Graubard BI, Andreotti G, Younes N, Cleary SD, Cook MB (2017). Prediagnostic body mass index trajectories in relation to prostate cancer incidence and mortality in the PLCO Cancer Screening Trial. J Natl Cancer Inst.

