# Supplementary material for: Tall height and obesity are associated with an increased risk of aggressive prostate cancer: results from the EPIC cohort study
Source: BMC Med. 2017 Jul 13;15:115. doi: 10.1186/s12916-017-0876-7 (PMC5508687; doi:10.1186/s12916-017-0876-7)
Supplement: Additional file 1: Table S1. — List of all of the local ethics committees for the European Prospective Investigation into Cancer and Nutrition (EPIC) study. Table S2. Distribution of cases by tumour grade in men from the EPIC study. Table S3. Baseline characteristics of participants according to fifths of height at recruitment in men from the EPIC study. Table S4. Baseline characteristics of participants according to fifths of waist circumference at recruitment in men from the EPIC study. Table S5. Distribution of study participants and prostate cancer cases by country. Table S6. Multivariable-adjusted hazard ratios (95% CI) for prostate cancer in relation to hip circumference and waist to hip ratio (WHR) at recruitment in men from the EPIC study. Table S7. Multivariable-adjusted hazard ratios (95% CI) for prostate cancer in relation to BMI, waist circumference and WHR using the WHO cut-off points at recruitment in men from the EPIC study. Table S8. Stratified and sensitivity analyses. Multivariable-adjusted hazard ratios (95% CI) for high-grade prostate cancer and death from prostate cancer in relation to height (per 10 cm unit increase) at recruitment in men from the EPIC study. Table S9. Stratified and sensitivity analyses. Multivariable-adjusted hazard ratios (95% CI) for high-grade prostate cancer and death from prostate cancer in relation to BMI (per 5 kg/m2 unit increase) at recruitment in men from the EPIC study. Table S10. Stratified and sensitivity analyses. Multivariable-adjusted hazard ratios (95% CI) for high-grade prostate cancer and death from prostate cancer in relation to waist circumference (per 10 cm unit increase) at recruitment in men from the EPIC study. (DOCX 63 kb) [file 12916_2017_876_MOESM1_ESM.docx]

**Additional file 1**

| **Table S1.** List of all of the local ethics committees for the EPIC study | |
| --- | --- |
| **Centre** | **Full Name of Ethics Committee or Institutional Review Board** |
| Athens | The Committee of Bioethics and Deontology of the Hellenic Health Foundation |
| Cambridge | Norwich District Ethics Committee |
| Denmark (Aarhus, Copenhagen) | The National Committee on Health Research Ethics |
| Heidelberg | Ethics Committee of the Heidelberg University Medical School |
| IARC | IARC Ethics Committee |
| Imperial College | Imperial College Research Ethics Committee [ICREC] |
| Italy (Milan, Ragusa, Turin) | Comitato Etico Indipendente, Fondazione IRCCS Istituto Nazionale dei Tumori, Milano |
| Florence | Comitato Etico Locale Azienda Sanitaria di Firenze |
| Malmo | Ethics Committee of Lund University |
| Netherlands (Bilthoven) | The Medical Ethical Committee (METC= Medisch Ethische Toetsingscommissie) of the University Medical Center Utrecht (UMCU), Utrecht, the Netherlands |
| Oxford | Scotland A Research Ethics Committee |
| Potsdam | Ethikkommission der Landesärztekammer Brandenburg Cottbus, Deutschland |
| Spain (Asturias, Barcelona, Granada, Murcia, Navarre, San Sebastian) | CEIC Comité de Ética de Investigación Clínica |
| Turin | Human Genetics Foundation Torino: Ethics Committee |
| Umea | Umea Regional Ethical Review Board |

| **Table S2.** Distribution of cases by tumour grade in men from the European Prospective Investigation into Cancer and Nutrition (EPIC) study. | | |
| --- | --- | --- |
| **Grade** | **Gleason sum** | **Prostate cancer cases, n** |
| Low-intermediate | 2 | 19 |
|  | 3 | 38 |
|  | 4 | 157 |
|  | 5 | 284 |
|  | 6 | 1393 |
|  | 7 | 1358 |
| High | 8 | 413 |
|  | 9 | 277 |
|  | 10 | 33 |
|  | **Grade as coded in the recruitment centre** |  |
| Low-intermediate | Well differentiated | 105 |
|  | Moderately differentiated | 288 |
|  | Poorly differentiated | 107 |
| High | Undifferentiated | 3 |
| Some people had both Gleason sum and grade as coded in the recruitment centre and when both were available we used Gleason sum. | | |

| **Table S3.** Baseline characteristics of participants according to fifths of height at recruitment in men from the European Prospective Investigation into Cancer and Nutrition (EPIC) study. | | | | | |
| --- | --- | --- | --- | --- | --- |
|  | **Height** | | | | |
| **Characteristic** | **1** | **2** | **3** | **4** | **5** |
| Number of men | 50678 | 68736 | 21698 | 21699 | 21700 |
| Age at baseline^1^, y | 54.6 (9.5) | 52.9 (9.5) | 52.0 (9.8) | 50.6 (10.1) | 48.2 (10.6) |
| Age at diagnosis^1^, y | 68.5 (7.0) | 68.5 (6.7) | 67.7 (6.4) | 67.7 (6.5) | 66.7 (6.3) |
| Smoking status, N (%) |  |  |  |  |  |
| Never smoker | 8552 (30.1) | 9662 (30.9) | 8016 (31.9) | 10500 (34.4) | 9853 (37.8) |
| Former smoker | 10448 (36.7) | 11647 (37.3) | 9457 (37.6) | 11028 (36.1) | 8755 (33.6) |
| Current smoker | 9022 (31.7) | 9422 (30.2) | 7323 (29.1) | 8673 (28.4) | 7204 (27.6) |
| Unknown | 433 (1.5) | 515 (1.6) | 361 (1.4) | 352 (1.2) | 282 (1.1) |
| Physical activity, N (%) |  |  |  |  |  |
| Inactive | 6946 (24.4) | 6126 (19.6) | 4478 (17.8) | 5056 (16.5) | 3941 (15.1) |
| Moderately inactive | 8114 (28.5) | 9502 (30.4) | 7777 (30.9) | 9698 (31.7) | 8190 (31.4) |
| Moderately active | 6791 (23.9) | 7548 (24.2) | 6157 (24.5) | 7483 (24.5) | 6380 (24.5) |
| Active | 6225 (21.9) | 7509 (24.0) | 6173 (24.5) | 7624 (25.0) | 6723 (25.8) |
| Unknown | 379 (1.3) | 561 (1.8) | 572 (2.3) | 692 (2.3) | 860 (3.3) |
| Diabetes at baseline, N (%) |  |  |  |  |  |
| No | 26425 (92.9) | 29357 (94.0) | 23718 (94.3) | 28922 (94.7) | 24901 (95.4) |
| Yes | 1665 (5.9) | 1220 (3.9) | 820 (3.3) | 826 (2.7) | 542 (2.1) |
| Unknown | 365 (1.3) | 669 (2.1) | 619 (2.5) | 805 (2.6) | 651 (2.5) |
| Education, N (%) |  |  |  |  |  |
| Below degree level | 23614 (83.0) | 23421 (75.0) | 17545 (69.7) | 20066 (65.7) | 15628 (59.9) |
| Degree level | 4080 (14.3) | 6943 (22.2) | 6867 (27.3) | 9682 (31.7) | 9869 (37.8) |
| Unknown | 761 (2.7) | 882 (2.8) | 745 (3.0) | 805 (2.6) | 597 (2.3) |
| Marital status, N (%) |  |  |  |  |  |
| Married | 15284 (53.7) | 17596 (56.3) | 14213 (56.5) | 17520 (57.3) | 14519 (55.6) |
| Not married | 2566 (9.0) | 3681 (11.8) | 3168 (12.6) | 4419 (14.5) | 4694 (18.0) |
| Unknown | 10605 (37.3) | 9969 (31.9) | 7776 (30.9) | 8614 (28.2) | 6881 (26.4) |
| Height^1^, cm | 164.4 (3.6) | 171.1 (1.3) | 174.9 (1.0) | 178.8 (1.3) | 185.3 (3.5) |
| BMI^1^, kg/m^2^ | 27.5 (3.8) | 26.8 (3.6) | 26.4 (3.5) | 26.1 (3.5) | 25.6 (3.5) |
| Weight^1^, kg | 74.2 (10.4) | 78.4 (10.6) | 80.9 (10.9) | 83.4 (11.4) | 88.1 (12.3) |
| Waist circumference^1^, cm | 94.7 (10.3) | 94.5 (10.2) | 94.5 (10.2) | 94.7 (10.3) | 95.1 (10.3) |
| Hip circumference^1^, cm | 99.7 (7.3) | 100.3 (6.9) | 100.8 (6.7) | 101.4 (6.6) | 102.7 (6.6) |
| WHR^1^ | 0.950 (0.063) | 0.942 (0.064) | 0.938 (0.063) | 0.934 (0.065) | 0.927 (0.065) |
| BMI, body mass index; WHR, waist to hip ratio.  ^1^ Values are means (SD). | | | | | |

| **Table S4.** Baseline characteristics of participants according to fifths of waist circumference at recruitment in men from the European Prospective Investigation into Cancer and Nutrition (EPIC) study. | | | | | |
| --- | --- | --- | --- | --- | --- |
|  | **Waist circumference** | | | | |
| **Characteristic** | **1** | **2** | **3** | **4** | **5** |
| Number of men | 25938 | 25357 | 25804 | 27793 | 23719 |
| Age at baseline^1^, y | 48.4 (11.5) | 51.6 (10.3) | 53.0 (9.0) | 54.0 (8.7) | 54.8 (8.5) |
| Age at diagnosis^1^, y | 67.4 (7.0) | 68.2 (7.0) | 68.0 (6.4) | 68.3 (6.3) | 68.3 (6.5) |
| Smoking status, N (%) |  |  |  |  |  |
| Never smoker | 9821 (37.9) | 8712 (34.4) | 7694 (29.8) | 7474 (26.9) | 5766 (24.3) |
| Former smoker | 7465 (28.8) | 9030 (35.6) | 9929 (38.5) | 11587 (41.7) | 10224 (43.1) |
| Current smoker | 8361 (32.2) | 7254 (28.6) | 7810 (30.3) | 8374 (30.1) | 7392 (31.2) |
| Unknown | 291 (1.1) | 361 (1.4) | 371 (1.4) | 358 (1.3) | 337 (1.4) |
| Physical activity, N (%) |  |  |  |  |  |
| Inactive | 3373 (13.0) | 3917 (15.4) | 4642 (18.0) | 5794 (20.8) | 6312 (26.6) |
| Moderately inactive | 7173 (27.7) | 7795 (30.7) | 8009 (31.0) | 8865 (31.9) | 7269 (30.6) |
| Moderately active | 6425 (24.8) | 6351 (25.0) | 6281 (24.3) | 6475 (23.3) | 5203 (21.9) |
| Active | 7991 (30.8) | 6680 (26.3) | 6333 (24.5) | 6156 (22.1) | 4551 (19.2) |
| Unknown | 976 (3.8) | 614 (2.4) | 539 (2.1) | 503 (1.8) | 384 (1.6) |
| Diabetes at baseline, N (%) |  |  |  |  |  |
| No | 24886 (95.9) | 24159 (95.3) | 24379 (94.5) | 25917 (93.3) | 21341 (90.0) |
| Yes | 483 (1.9) | 604 (2.4) | 813 (3.2) | 1227 (4.4) | 1750 (7.4) |
| Unknown | 569 (2.2) | 594 (2.3) | 612 (2.4) | 649 (2.3) | 628 (2.6) |
| Education, N (%) |  |  |  |  |  |
| Below degree level | 16014 (61.7) | 16306 (64.3) | 17986 (69.7) | 20661 (74.3) | 18740 (79.0) |
| Degree level | 9113 (35.1) | 8156 (32.2) | 7070 (27.4) | 6424 (23.1) | 4427 (18.7) |
| Unknown | 811 (3.1) | 895 (3.5) | 748 (2.9) | 708 (2.5) | 552 (2.3) |
| Marital status, N (%) |  |  |  |  |  |
| Married | 14616 (56.3) | 14641 (57.7) | 13945 (54.0) | 14067 (50.6) | 11520 (48.6) |
| Not married | 5759 (22.2) | 3408 (13.4) | 2477 (9.6) | 2407 (8.7) | 2093 (8.8) |
| Unknown | 5563 (21.4) | 7308 (28.8) | 9382 (36.4) | 11319 (40.7) | 10106 (42.6) |
| Height^1^, cm | 174.2 (7.3) | 174.4 (7.2) | 174.4 (7.3) | 174.4 (7.4) | 174.5 (7.6) |
| BMI^1^, kg/m^2^ | 22.7 (2.0) | 24.8 (1.8) | 26.3 (1.8) | 28.0 (2.0) | 31.5 (3.2) |
| Weight^1^, kg | 68.9 (7.3) | 75.5 (6.6) | 79.9 (6.8) | 85.1 (7.5) | 96.0 (11.2) |
| Waist circumference^1^, cm | 81.3 (4.1) | 89.0 (1.5) | 94.0 (1.4) | 99.7 (1.9) | 110.2 (6.5) |
| Hip circumference^1^, cm | 94.0 (4.5) | 97.8 (3.9) | 100.2 (4.0) | 103.3 (4.3) | 109.3 (6.5) |
| WHR^1^ | 0.866 (0.046) | 0.913 (0.038) | 0.940 (0.039) | 0.967 (0.040) | 1.010 (0.052) |
| BMI, body mass index; WHR, waist to hip ratio.  ^1^ Values are means (SD). | | | | | |

| **Table S5**. Distribution of the study participants and of prostate cancer cases by country. | | | | |
| --- | --- | --- | --- | --- |
| **Country** | **All participants, n (%)** | **Prostate cancer cases, n (%)** | **Height, m (SD)** | **BMI, kg/m^2^ (SD)** |
| Denmark | 26,280 (18.5) | 1,884 (26.8) | 176.7 (6.5) | 26.6 (3.6) |
| Germany | 21,149 (14.9) | 832 (11.8) | 175.4 (6.7) | 27.0 (3.6) |
| Greece | 10,782 (7.6) | 95 (1.4) | 170.0 (7.1) | 27.8 (3.8) |
| Italy | 13,912 (9.8) | 477 (6.8) | 171.7 (6.8) | 26.4 (3.4) |
| The Netherlands | 9,626 (6.8) | 215 (3.1) | 178.4 (7.3) | 25.5 (3.5) |
| Spain | 15,094 (10.6) | 666 (9.5) | 169.0 (6.3) | 28.4 (3.4) |
| Sweden | 22,245 (15.7) | 1,830 (26.1) | 177.3 (6.7) | 25.6 (3.5) |
| United Kingdom | 22,808 (16.1) | 1,025 (14.6) | 175.5 (6.8) | 25.4 (3.5) |
| All countries | 141,896 (100.0) | 7,024 (100.0) | 174.7 (7.4) | 26.5 (3.7) |

| **Table S6.** Multivariable-adjusted hazard ratios (95 % CI) for prostate cancer in relation to hip circumference and WHR at recruitment in men from the European Prospective Investigation into Cancer and Nutrition (EPIC) study. | | | | | | | | | |
| --- | --- | --- | --- | --- | --- | --- | --- | --- | --- |
|  | **No. of cases** | **Fifths** | | | | | ***P* for trend**^1^ | ***P* for het.**^2^ |  |
|  |  | **1** | **2** | **3** | **4** | **5** |  |  |  |
|  |  | **HR** | **HR (95 % CI)** | **HR (95 % CI)** | **HR (95 % CI)** | **HR (95 % CI)** |  |  |  |
| **Hip, cm** |  |  |  |  |  |  |  |  | **Continuous per 10 cm** |
| Median (range) |  | 93.0 (50.0 - 95.2) | 97.5 (95.2 - 99.0) | 101.0 (99.0 - 102.0) | 104.0 (102.0 - 106.0) | 110.0 (106.0 - 180.0) |  |  |  |
| Total PCa | 6321 | 1.00 ref | 0.93 (0.87 - 1.01) | 0.95 (0.88 - 1.03) | 0.95 (0.88 - 1.03) | 0.86 (0.79 - 0.94) | 0.001 |  | 0.93 (0.89 - 0.98) |
| Grade |  |  |  |  |  |  |  |  |  |
| Low-intermediate | 3234 | 1.00 ref | 0.95 (0.85 - 1.05) | 0.93 (0.83 - 1.04) | 0.92 (0.82 - 1.03) | 0.84 (0.75 - 0.95) | 0.001 |  | 0.91 (0.85 - 0.97) |
| High | 639 | 1.00 ref | 1.14 (0.88 - 1.48) | 1.22 (0.94 - 1.60) | 1.39 (1.07 - 1.81) | 1.37 (1.04 - 1.80) | 0.03 | <0.001 | 1.21 (1.05 - 1.40) |
| Stage |  |  |  |  |  |  |  |  |  |
| Localized | 2075 | 1.00 ref | 0.92 (0.80 - 1.05) | 0.98 (0.85 - 1.13) | 0.91 (0.79 - 1.05) | 0.90 (0.77 - 1.04) | 0.03 |  | 0.95 (0.87 - 1.03) |
| Advanced | 1288 | 1.00 ref | 1.02 (0.86 - 1.21) | 0.98 (0.82 - 1.17) | 1.04 (0.87 - 1.24) | 0.97 (0.81 - 1.17) | 0.8 | 0.5 | 0.99 (0.89 - 1.09) |
|  |  |  |  |  |  |  |  |  |  |
| PCa death | 870 | 1.00 ref | 1.04 (0.84 - 1.30) | 1.23 (0.99 - 1.53) | 1.21 (0.96 - 1.51) | 1.43 (1.14 - 1.79) | 0.001 |  | 1.24 (1.10 - 1.40) |
| **WHR** |  |  |  |  |  |  |  |  | **Continuous per 0.1 unit** |
| Median (range) |  | 0.86 (0.35 - 0.88) | 0.91 (0.89 - 0.92) | 0.94 (0.93 - 0.94) | 0.97 (0.95 - 0.99) | 1.02 (1.00 - 1.90) |  |  |  |
| Total PCa | 6321 | 1.00 ref | 0.97 (0.90 - 1.06) | 0.96 (0.88 - 1.06) | 0.96 (0.89 - 1.04) | 0.97 (0.89 - 1.06) | 0.5 |  | 0.98 (0.93 - 1.03) |
| Grade |  |  |  |  |  |  |  |  |  |
| Low-intermediate | 3234 | 1.00 ref | 0.94 (0.84 - 1.06) | 0.93 (0.82 - 1.06) | 0.96 (0.85 - 1.07) | 0.92 (0.81 - 1.04) | 0.1 |  | 0.96 (0.90 - 1.04) |
| High | 639 | 1.00 ref | 1.09 (0.82 - 1.45) | 1.40 (1.04 - 1.88) | 1.23 (0.94 - 1.62) | 1.46 (1.09 - 1.94) | 0.004 | 0.004 | 1.25 (1.06 - 1.47) |
| Stage |  |  |  |  |  |  |  |  |  |
| Localized | 2075 | 1.00 ref | 0.94 (0.81 - 1.09) | 0.90 (0.76 - 1.07) | 0.91 (0.79 - 1.05) | 0.93 (0.79 - 1.08) | 0.2 |  | 0.96 (0.87 - 1.05) |
| Advanced | 1288 | 1.00 ref | 1.20 (0.99 - 1.46) | 1.07 (0.85 - 1.33) | 1.21 (1.00 - 1.46) | 1.29 (1.05 - 1.58) | 0.01 | 0.02 | 1.14 (1.02 - 1.28) |
|  |  |  |  |  |  |  |  |  |  |
| PCa death | 870 | 1.00 ref | 0.94 (0.75 - 1.19) | 0.94 (0.73 - 1.23) | 1.03 (0.82 - 1.28) | 1.19 (0.94 - 1.50) | 0.003 |  | 1.14 (0.99 - 1.31) |
| PCa, prostate cancer; WHR, waist to hip ratio.  Cox regression analysis. All models are stratified by centre and age at recruitment and adjusted for education level (less than university, university graduate, missing), smoking status (never, former, current, missing), marital status (married, not married, missing), diabetes (yes, no, missing), and physical activity (inactive, moderately inactive, moderately active, active, missing).  ^1^ *P*-values for trend are obtained by entering the continuous anthropometric variable in the model.  ^2^ *P*-value from test for heterogeneity for the associations of height with risk of prostate cancer categorized according to prostate tumour grade (low-intermediate or high) and stage (localized or advanced).  Low-intermediate grade (Gleason score of <8, or grade coded as well, moderately, or poorly differentiated). High grade (Gleason score of ≥ 8, or grade coded as undifferentiated). Localized stage (TNM staging score of ≤T_2_ and N_0_/N_x_ and M_0_, or stage coded in the recruitment center as localized). Advanced stage (T_3_-T_4_ and/or N_1_-N_3_ and/or M_1_, and/or stage coded in the recruitment center as metastatic). | | | | | | | | | |

| **Table S7.** Multivariable-adjusted hazard ratios (95 % CI) for prostate cancer in relation to BMI, waist circumference and WHR using the WHO cut-off points at recruitment in men from the European Prospective Investigation into Cancer and Nutrition (EPIC) study. | | | | | | | | | | | |
| --- | --- | --- | --- | --- | --- | --- | --- | --- | --- | --- | --- |
|  | **BMI (kg/m^2^)** | | |  | **Waist circumference (cm)** | | |  | **WHR** | |  |
|  | **<25** | **25-29.9** | **≥30** | ***P* for trend**^1^ | **<94** | **94-101.9** | **≥ 102 (higher risk)** | ***P* for trend**^1^ | **<0.90** | **≥ 0.90** | ***P* for trend**^1^ |
| n | 50,678 | 68,736 | 21,698 |  | 62,459 | 36,499 | 29,653 |  | 29,939 | 96,276 |  |
| Total PCa | 1.00 ref | 0.98 (0.93 - 1.03) | 0.89 (0.82 - 0.96) | <0.001 | 1.00 ref | 0.99 (0.93 - 1.05) | 0.93 (0.87 - 1.00) | 0.01 | 1.00 ref | 1.00 (0.94 - 1.07) | 0.5 |
| Grade |  |  |  |  |  |  |  |  |  |  |  |
| Low-intermediate | 1.00 ref | 1.00 (0.92 - 1.07) | 0.87 (0.78 - 0.97) | 0.008 | 1.00 ref | 0.96 (0.89 - 1.05) | 0.91 (0.83 - 1.00) | 0.03 | 1.00 ref | 0.96 (0.88 - 1.05) | 0.06 |
| High | 1.00 ref | 1.06 (0.89 - 1.25) | 1.09 (0.86 - 1.38) | 0.1 | 1.00 ref | 1.26 (1.04 - 1.52) | 1.24 (1.02 - 1.53) | 0.003 | 1.00 ref | 1.36 (1.09 - 1.70) | 0.06 |
| Stage |  |  |  |  |  |  |  |  |  |  |  |
| Localized | 1.00 ref | 0.94 (0.86 - 1.02) | 0.84 (0.74 - 0.95) | 0.001 | 1.00 ref | 0.94 (0.85 - 1.04) | 0.90 (0.81 - 1.01) | 0.03 | 1.00 ref | 0.96 (0.86 - 1.08) | 0.2 |
| Advanced | 1.00 ref | 1.12 (0.99 - 1.26) | 1.05 (0.88 - 1.24) | 0.3 | 1.00 ref | 1.14 (1.00 - 1.30) | 1.02 (0.88 - 1.19) | 0.2 | 1.00 ref | 1.17 (1.01 - 1.37) | 0.01 |
|  |  |  |  |  |  |  |  |  |  |  |  |
| PCa death | 1.00 ref | 1.04 (0.89 - 1.20) | 1.29 (1.06 - 1.58) | 0.01 | 1.00 ref | 1.22 (1.04 - 1.43) | 1.35 (1.14 - 1.60) | <0.001 | 1.00 ref | 1.08 (0.90 - 1.29) | 0.003 |
| BMI, body mass index; PCa, prostate cancer; WHR, waist to hip ratio.  Cox regression analysis. All models are stratified by centre and age at recruitment and adjusted for education level (less than university, university graduate, missing), smoking status (never, former, current, missing), marital status (married, not married, missing), diabetes (yes, no, missing), and physical activity (inactive, moderately inactive, moderately active, active, missing).  ^1^ *P*-values for trend are obtained by entering the continuous anthropometric variable in the model.  ^2^ *P*-value from test for heterogeneity for the associations of each anthropometric variable with risk of prostate cancer categorized according to prostate tumour grade (low-intermediate or high) and stage (localized or advanced).  Low-intermediate grade (Gleason score of <8, or grade coded as well, moderately, or poorly differentiated). High grade (Gleason score of ≥ 8, or grade coded as undifferentiated). Localized stage (TNM staging score of ≤T_2_ and N_0_/N_x_ and M_0_, or stage coded in the recruitment center as localized). Advanced stage (T_3_-T_4_ and/or N_1_-N_3_ and/or M_1_, and/or stage coded in the recruitment center as metastatic). | | | | | | | | | | | |

| **Table S8.** Stratified and sensitivity analyses. Multivariable-adjusted hazard ratios (95 % CI) for high grade prostate cancer and death from prostate cancer in relation to height (per 10 cm unit increase) at recruitment in men from the European Prospective Investigation into Cancer and Nutrition (EPIC) study. | | | | | | |
| --- | --- | --- | --- | --- | --- | --- |
|  | **High grade PCa** | | | **PCa death** | | |
| **Factor and subset** | **Cases** | **HR (95% CI)**^1^ | ***P* for het.** | **Cases** | **HR (95% CI)**^1^ | ***P* for het.** |
| Overall | 726 | 1.21 (1.06 - 1.38) |  | 932 | 1.17 (1.04 - 1.31) |  |
| Age at recruitment, y |  |  |  |  |  |  |
| <60 | 405 | 1.22 (1.03 - 1.45) |  | 355 | 1.11 (0.92 - 1.33) |  |
| ≥60 | 321 | 1.20 (0.99 - 1.45) | 0.6^2^ | 577 | 1.20 (1.04 - 1.39) | 0.7^2^ |
| Age at diagnosis, y |  |  |  |  |  |  |
| <65 | 204 | 1.25 (0.98 - 1.59) |  | 259 | 1.01 (0.81 - 1.24) |  |
| ≥65 | 522 | 1.20 (1.03 - 1.39) | 0.8^3^ | 673 | 1.23 (1.08 - 1.41) | 0.1^3^ |
| Time between recruitment and diagnosis (or death) |  |  |  |  |  |  |
| <5 years | 76 | 1.16 (0.78 - 1.71) |  | 284 | 1.00 (0.81 - 1.22) |  |
| ≥5 years | 650 | 1.22 (1.06 - 1.39) | 0.8^3^ | 648 | 1.25 (1.09 - 1.43) | 0.07^3^ |
| Excluding extreme height values: percentiles 1–99 | 715 | 1.23 (1.07 - 1.40) |  | 912 | 1.18 (1.05 - 1.32) |  |
| Additionally adjusted for total energy intake | 726 | 1.20 (1.06 - 1.37) |  | 932 | 1.16 (1.03 - 1.29) |  |
| Additionally adjusted for total vegetables consumption | 726 | 1.21 (1.06 - 1.37) |  | 932 | 1.16 (1.04 - 1.30) |  |
| Additionally adjusted for total fruit consumption | 726 | 1.21 (1.07 - 1.38) |  | 932 | 1.17 (1.04 - 1.31) |  |
| Additionally adjusted for alcohol consumption | 726 | 1.21 (1.06 - 1.37) |  | 932 | 1.17 (1.04 - 1.31) |  |
| Additionally adjusted for red meat consumption | 647 | 1.27 (1.11 - 1.46) |  | 878 | 1.18 (1.05 - 1.32) |  |
| Additionally adjusted for processed meat consumption | 726 | 1.21 (1.06 - 1.38) |  | 932 | 1.16 (1.04 - 1.30) |  |
| Additionally adjusted for protein from dairy consumption | 726 | 1.21 (1.06 - 1.37) |  | 932 | 1.16 (1.04 - 1.30) |  |
| Excluding men with missing values for any of the covariates | 353 | 1.13 (0.94 - 1.36) |  | 455 | 1.11 (0.94 - 1.31) |  |
| PCa, prostate cancer.  Cox regression analysis. All models are stratified by centre and age at recruitment and adjusted for education level (less than university, university graduate, missing), smoking status (never, former, current, missing), marital status (married, not married, missing), diabetes (yes, no, missing), and physical activity (inactive, moderately inactive, moderately active, active, missing), unless otherwise specified.  ^1^ HR (95% CI) estimated per 10 cm unit increase in height.  ^2^ *P*-value for heterogeneity based on likelihood ratio test to compare the Cox models with and without interactions terms of height with age at recruitment.  ^3^ *P*-value for heterogeneity based on the difference in the association of height with prostate cancer death risk between subgroups.  Low-intermediate grade (Gleason score of <8, or grade coded as well, moderately, or poorly differentiated). High grade (Gleason score of ≥ 8, or grade coded as undifferentiated). Localized stage (TNM staging score of ≤T_2_ and N_0_/N_x_ and M_0_, or stage coded in the recruitment center as localized). Advanced stage (T_3_-T_4_ and/or N_1_-N_3_ and/or M_1_, and/or stage coded in the recruitment center as metastatic). | | | | | | |

| **Table S9**. Stratified and sensitivity analyses. Multivariable-adjusted hazard ratios (95 % CI) for high grade prostate cancer and death from prostate cancer in relation to BMI (per 5 kg/m^2^ unit increase) at recruitment in men from the European Prospective Investigation into Cancer and Nutrition (EPIC) study. | | | | | | |
| --- | --- | --- | --- | --- | --- | --- |
|  | **High grade PCa** | | | **PCa death** | | |
| **Factor and subset** | **Cases** | **HR (95% CI)**^1^ | ***P* for het.** | **Cases** | **HR (95% CI)**^1^ | ***P* for het.** |
| Overall | 720 | 1.10 (0.97 - 1.25) |  | 931 | 1.14 (1.02 - 1.27) |  |
| Age at recruitment, y |  |  |  |  |  |  |
| <60 | 401 | 1.04 (0.88 - 1.24) |  | 355 | 1.12 (0.94 - 1.35) |  |
| ≥60 | 319 | 1.18 (0.97 - 1.43) | 0.4**^2^** | 576 | 1.15 (1.00 - 1.33) | 0.6**^2^** |
| Age at diagnosis, y |  |  |  |  |  |  |
| <65 | 203 | 1.11 (0.86 - 1.42) |  | 259 | 1.09 (0.88 - 1.35) |  |
| ≥65 | 517 | 1.13 (0.95 - 1.33) | 0.9^3^ | 672 | 1.24 (1.08 - 1.43) | 0.3^3^ |
| Time between recruitment and diagnosis (or death) |  |  |  |  |  |  |
| <5 years | 75 | 0.94 (0.63 - 1.40) |  | 284 | 1.07 (0.87 - 1.31) |  |
| ≥5 years | 645 | 1.12 (0.98 - 1.29) | 0.4^3^ | 647 | 1.17 (1.02 - 1.34) | 0.4^3^ |
| Excluding extreme BMI values: percentiles 1–99 | 711 | 1.22 (0.94 - 1.60) |  | 913 | 1.32 (1.04 - 1.67) |  |
| Additionally adjusted for total energy intake | 720 | 1.10 (0.97 - 1.26) |  | 931 | 1.14 (1.02 - 1.28) |  |
| Additionally adjusted for total vegetables consumption | 720 | 1.10 (0.97 - 1.25) |  | 931 | 1.14 (1.02 - 1.28) |  |
| Additionally adjusted for total fruit consumption | 720 | 1.10 (0.97 - 1.25) |  | 931 | 1.14 (1.02 - 1.28) |  |
| Additionally adjusted for alcohol consumption | 720 | 1.10 (0.97 - 1.25) |  | 931 | 1.13 (1.01 - 1.27) |  |
| Additionally adjusted for red meat consumption | 641 | 1.16 (1.01 - 1.33) |  | 877 | 1.16 (1.03 - 1.30) |  |
| Additionally adjusted for processed meat consumption | 720 | 1.10 (0.96 - 1.25) |  | 931 | 1.13 (1.01 - 1.27) |  |
| Additionally adjusted for protein from dairy consumption | 720 | 1.10 (0.97 - 1.25) |  | 931 | 1.14 (1.02 - 1.27) |  |
| Additionally adjusted for height | 720 | 1.11 (0.98 - 1.27) |  | 931 | 1.15 (1.03 - 1.29) |  |
| Excluding men with missing values for any of the covariates | 349 | 1.05 (0.87 - 1.26) |  | 455 | 1.04 (0.88 - 1.23) |  |
| BMI adjusted for waist circumference (residuals)^4^ | 635 | 0.99 (0.77 - 1.29) |  | 874 | 0.89 (0.72 - 1.12) |  |
| BMI, body mass index PCa, prostate cancer.  Cox regression analysis. All models are stratified by centre and age at recruitment and adjusted for education level (less than university, university graduate, missing), smoking status (never, former, current, missing), marital status (married, not married, missing), diabetes (yes, no, missing), and physical activity (inactive, moderately inactive, moderately active, active, missing), unless otherwise specified.  ^1^ HR (95% CI) estimated per 5 kg/m^2^ unit increase in BMI.  ^2^ *P*-value for heterogeneity based on likelihood ratio test to compare the Cox models with and without interactions terms of BMI with age at recruitment.  ^3^ *P*-value for heterogeneity based on the difference in the association of BMI with prostate cancer death risk between subgroups.  ^4^ HR (95% CI) is from the multiple adjusted model (above) after accounting for the residuals of BMI regressed on waist circumference.  Low-intermediate grade (Gleason score of <8, or grade coded as well, moderately, or poorly differentiated). High grade (Gleason score of ≥ 8, or grade coded as undifferentiated). Localized stage (TNM staging score of ≤T_2_ and N_0_/N_x_ and M_0_, or stage coded in the recruitment center as localized). Advanced stage (T_3_-T_4_ and/or N_1_-N_3_ and/or M_1_, and/or stage coded in the recruitment center as metastatic). | | | | | | |

| **Table S10.** Stratified and sensitivity analyses. Multivariable-adjusted hazard ratios (95 % CI) for high grade prostate cancer and death from prostate cancer in relation to waist circumference (per 10 cm unit increase) at recruitment in men from the European Prospective Investigation into Cancer and Nutrition (EPIC) study. | | | | | | |
| --- | --- | --- | --- | --- | --- | --- |
|  | **High grade PCa** | | | **PCa death** | | |
| **Factor and subset** | **Cases** | **HR (95% CI)**^1^ | ***P* for het.** | **Cases** | **HR (95% CI)**^1^ | ***P* for het.** |
| Overall | 641 | 1.13 (1.03 - 1.25) |  | 877 | 1.18 (1.08 - 1.28) |  |
| Age at recruitment, y |  |  |  |  |  |  |
| <60 | 350 | 1.13 (0.99 - 1.29) |  | 326 | 1.25 (1.09 - 1.43) |  |
| ≥60 | 291 | 1.15 (0.99 - 1.33) | 0.9^2^ | 551 | 1.14 (1.02 - 1.26) | 0.3^2^ |
| Age at diagnosis, y |  |  |  |  |  |  |
| <65 | 183 | 1.09 (0.91 - 1.31) |  | 238 | 1.10 (0.94 - 1.28) |  |
| ≥65 | 458 | 1.15 (1.02 - 1.29) | 0.6^3^ | 639 | 1.21 (1.10 - 1.33) | 0.3^3^ |
| Time between recruitment and diagnosis (or death) |  |  |  |  |  |  |
| <5 years | 75 | 1.07 (0.81 - 1.42) |  | 270 | 1.14 (0.98 - 1.32) |  |
| ≥5 years | 566 | 1.14 (1.03 - 1.27) | 0.7^3^ | 607 | 1.20 (1.08 - 1.32) | 0.3^3^ |
| Excluding extreme waist values: percentiles 1–99 | 629 | 1.14 (1.03 - 1.27) |  | 862 | 1.19 (1.09 - 1.30) |  |
| Additionally adjusted for total energy intake | 641 | 1.13 (1.02 - 1.25) |  | 877 | 1.18 (1.08 - 1.28) |  |
| Additionally adjusted for total vegetables consumption | 641 | 1.13 (1.03 - 1.25) |  | 877 | 1.18 (1.08 - 1.28) |  |
| Additionally adjusted for total fruit consumption | 641 | 1.13 (1.03 - 1.25) |  | 877 | 1.18 (1.08 - 1.28) |  |
| Additionally adjusted for alcohol consumption | 641 | 1.13 (1.02 - 1.25) |  | 877 | 1.17 (1.08 - 1.27) |  |
| Additionally adjusted for red meat consumption | 641 | 1.13 (1.02 - 1.25) |  | 877 | 1.18 (1.08 - 1.28) |  |
| Additionally adjusted for processed meat consumption | 641 | 1.13 (1.02 - 1.25) |  | 877 | 1.17 (1.08 - 1.28) |  |
| Additionally adjusted for protein from dairy consumption | 641 | 1.13 (1.02 - 1.25) |  | 877 | 1.17 (1.08 - 1.28) |  |
| Additionally adjusted for height | 641 | 1.10 (1.00 - 1.22) |  | 875 | 1.16 (1.07 - 1.26) |  |
| Excluding men with missing values for any of the covariates | 271 | 1.19 (1.03 - 1.39) |  | 402 | 1.24 (1.10 - 1.39) |  |
| Waist circumference adjusted for BMI (residuals)^4^ | 635 | 1.13 (0.94 - 1.37) |  | 874 | 1.22 (1.04 - 1.44) |  |
| BMI, body mass index PCa, prostate cancer.  Cox regression analysis. All models are stratified by centre and age at recruitment and adjusted for education level (less than university, university graduate, missing), smoking status (never, former, current, missing), marital status (married, not married, missing), diabetes (yes, no, missing), and physical activity (inactive, moderately inactive, moderately active, active, missing), unless otherwise specified.  ^1^ HR (95% CI) estimated per 10 cm unit increase in waist circumference.  ^2^ *P*-value for heterogeneity based on likelihood ratio test to compare the Cox models with and without interactions terms of waist circumference with age at recruitment.  ^3^ *P*-value for heterogeneity based on the difference in the association of waist circumference with prostate cancer death risk between subgroups.  ^4^ HR (95% CI) is from the multiple adjusted model (above) after accounting for the residuals of waist circumference regressed on BMI.  Low-intermediate grade (Gleason score of <8, or grade coded as well, moderately, or poorly differentiated). High grade (Gleason score of ≥ 8, or grade coded as undifferentiated). Localized stage (TNM staging score of ≤T_2_ and N_0_/N_x_ and M_0_, or stage coded in the recruitment center as localized). Advanced stage (T_3_-T_4_ and/or N_1_-N_3_ and/or M_1_, and/or stage coded in the recruitment center as metastatic). | | | | | | |
